# Production of Low Molecular Weight Chitosan Using a Combination of Weak Acid and Ultrasonication Methods

**DOI:** 10.3390/polym14163417

**Published:** 2022-08-21

**Authors:** Suryani Suryani, Anis Yohana Chaerunisaa, I. Made Joni, Ruslin Ruslin, La Ode Ahmad Nur Ramadhan, Yoga Windhu Wardhana, Sitti Hadijah Sabarwati

**Affiliations:** 1 Doctor of Pharmacy Study Program, Faculty of Pharmacy, Universitas Padjadjaran, Sumedang 45363, Indonesia; 2 Department of Pharmacy, Faculty of Pharmacy, Universitas Halu Oleo, Kendari 93232, Indonesia; 3 Department of Pharmaceutics and Pharmaceutical Technology, Faculty of Pharmacy, Universitas Padjadjaran, Sumedang 45363, Indonesia; 4 Dosage Form Development Research Centre, Faculty of Pharmacy, Universitas Padjadjaran, Sumedang 45363, Indonesia; 5 Departement of Physics, Faculty of Mathematics and Natural Sciences, Universitas Padjadjaran, Sumedang 45363, Indonesia; 6 Functional Nano Powder University Centre of Excellence, Universitas Padjadjaran, Sumedang 45363, Indonesia; 7 Department of Chemistry, Faculty of Mathematics and Natural Science, Universitas Halu Oleo, Kendari 93232, Indonesia

**Keywords:** optimization, low molecular weight chitosan, depolymerization, Box-Behnken Design

## Abstract

Low molecular weight chitosan (LMWC) has higher solubility and lower viscosity allowing for a wider pharmaceutical application compared to high molecular weight chitosan. LMWC chitosan can be obtained through a chitosan depolymerization process. This research aimed to produce LWMC using the combination of formic acid and ultrasonication method with the optimal condition of the depolymerization process. The chitosan depolymerization method was performed by combining formic acid and ultrasonication. The optimum conditions of the depolymerization process were obtained using the Box–Behnken design. The LMWC obtained from depolymerization was characterized to identify its yield, degree of deacetylation, the molecular weight, structure, morphology, thermal behavior, and crystallinity index. Results: The characterization results of LWMC obtained from the depolymerization process using the optimum conditions showed that the yield was 89.398%; the degree of deacetylation was 98.076%; the molecular weight was 32.814 kDa; there was no change in the chemical structure, LWMC had disorganized shape, there was no change in the thermal behavior, and LWMC had a more amorphous shape compared to native chitosan. Conclusion: The production of LWMC involving depolymerization in the presence of weak acid and ultrasonication can be developed by using the optimal condition of the depolymerization process.

## 1. Introduction

Chitosan is a deacetylated product from chitin [1]. Chitin is present in the exoskeleton of crustaceans, insects, fungi, bacteria, and some mushrooms [2,3]. Chitosan is a functional natural polymer because of its properties of biodegradability [4], biocompatibility [5], bio adhesivity, non-toxicity [6], and it is able to form a gel and cationic at a low pH [2,7,8]. The molecular weight of chitosan is related to its properties [9]. The high molecular weight of chitosan provides low solubility, thus limiting its use [3]. Molecular weight affects not only the solubility of chitosan [10], but also its biodegradability, biocompatibility, bioactivity, and toxicity. Low molecular weight chitosan has better biodegradability, biocompatibility, and bioactivity [11] as well as lower toxicity [12] compared to high molecular weight chitosan. In addition, low molecular weight chitosan also has a higher solubility [13] and lower viscosity [14] due to the shorter chain and the presence of the free amine group on the glucosamine unit [15], allowing for a wider pharmaceutical application [16]. Low molecular weight chitosan has been used for drug delivery systems [17], demonstrating an increased swelling property makes chitosan a perfect carrier for the drug. It was reported that reducing the molecular weight of chitosan leads to reduce the viscosity of the chitosan solution, obtaining particles with a smaller size [18].

LMWC chitosan can be obtained through a chitosan depolymerization process [19]. Depolymerization of chitosan into low molecular weight chitosan can be done by chemical [20], enzymatic [21], and physical methods [22]. One of the chemical depolymerization methods is followed by using acids [23,24]. The acid method has advantages: it can be used for large-scale production, has a low cost of production [19], can produce smaller fragments in large quantities, and can increase the rate of chitosan hydrolysis. However, the use of strong acids with a high concentration may damage the environment [3]. Besides, the use of an excessive amount of acids in the depolymerization process causes glucosamine degradation of chitosan [25]. Additionally, chitosan depolymerization by strong acid is excluded from medical application due to its toxicity [26]. Using weak acids in depolymerization comes as an alternative to using strong acids in depolymerization. Using weak acids is considered safer to produce LMWC for pharmaceutical application than using strong acids for chitosan depolymerization. The utilization of very low concentration of weak acid for the degradation of chitosan was reported by Savitri et al. (2014). Formic acid is a weak acid that can be used in depolymerization. Formic acid depolymerization with the combination of H_2_O_2_ significantly decreases the molecular weight of chitosan [27]. In addition to the acid methods, physical methods [28] using ultrasonication can also lower the molecular weight of chitosan [29]. Depolymerization by sonication is more environmentally friendly, energy efficient [30], and effective [31]. The principle of the sonication method is to provide energy to break chemical bonds [29]. Characterization results show that depolymerization using ultrasonication does not change chitosan structure [32].

Optimization of a depolymerization process can be a complicated task [33], particularly when using a combination of 2 methods, namely formic acid and ultrasonication which responses were strongly affected by chitosan concentration, temperature, and time of sonication. The depolymerization process should be optimized to obtain the optimal condition of the depolymerization process that affect the responses [34]. The depolymerization process can be optimized by using response surface method (RSM) [33]. RSM is an efficient way to optimize the depolymerization process [35,36]. RSM is a multivariate statistical technique used to predict the optimum conditions of a process that can discover the best possible responses in the investigated experimental area [34,37]. RSM can predict the quadratic effect of the independent variable on the response through a mathematical model of the resulting quadratic equation [38]. The Box–Behnken design (BBD) is the main type of RSM [33]. The BBD only has three levels per factor [39,40] which are low (−1), middle (0), and high level (+1), respectively, and produce a minimum number of runs then BBD is less expensive to run than other RSM design [33,41].

**Figure 1:**
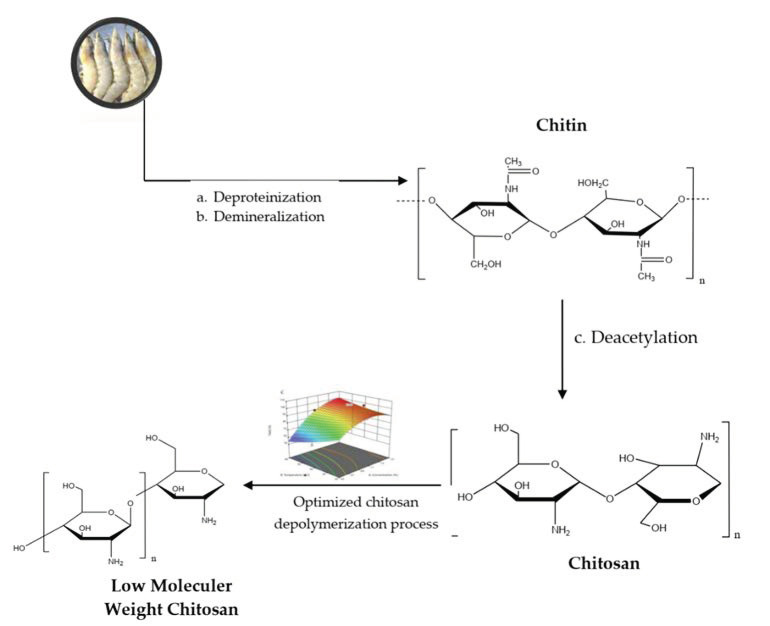
Strategy for production of low molecular weight chitosan by depolymerization.

Our objective was to produce low molecular weight chitosan involving depolymerization in the presence of weak acid (formic acid) and ultrasonication. The Box–Behnken design was used to optimize the parameters of the depolymerization process. The strategy for producing a low molecular weight of chitosan using this method is shown in Figure 1.

## 2. Materials and Methods

### 2.1. Materials

The materials of this research are shown in Table 1.

### 2.2. Shrimp Shell Preparation

A total of 10 kg of shrimp shell waste was washed with 50 L of water at room temperature for 1 h, then dried at 50 °C for 24 h in an oven (memmert, Schwabach, Germany). Clean shrimp shell waste was then mashed into powder. The powder was then sieved using a 20-mesh sieve.

### 2.3. Characterization of Shrimp Shell Powder

Shrimp powder was characterized by organoleptic tests [42,43], solubility in water [14], solubility in 95% ethanol [14], ash content (SNI 01-2354.1-2006), fat content (SNI 01-2354.3-2006), protein content (SNI 01 2354.4-2006), and water content (SNI 2354.2:2015).

### 2.4. Chitosan Preparation

#### 2.4.1. Deproteinization

A total of 500 g of shrimp shells were placed in a beaker glass equipped with a stirrer and thermometer (LT-12, Midwest Homebrewing, Philadelphia, PA, USA). Then, 3.5% NaOH was added with a ratio of shrimp shell mass to the solution of 1:10 (*w/v*) and heated on a hot plate (Stuart UC 152, Vernon Hills, IL, USA) for 2 h at a temperature of 65 °C with constant stirring. The precipitate was washed with distilled water until a neutral pH was achieved. The precipitate was dried in an oven at 60 °C for 24 h [44].

#### 2.4.2. Demineralization

The chitin resulting from the deproteinization was placed in a beaker glass equipped with a stirrer and a thermometer and placed on a hot plate. Then 1N HCl was added with a ratio of 1:15 (*w*/*v*) for 1 h at room temperature, and the precipitate was dried in an oven at 60 °C for 24 h [44].

#### 2.4.3. Deacetylation

The chitin obtained from the above processes was added to 50% NaOH solution with a ratio of chitin to the solution of 1:20 (*w*/*v*) at 120 °C while constantly stirred for 3 × 3 h. The deacetylation process aimed to produce a final product in the form of chitosan powder [44].

### 2.5. Chitosan Characterization Based on Pharmaceutical Requirement

The chitosan was then characterized in terms of its physicochemical properties to meet the requirements of pharmaceutical-grade chitosan, including organoleptic [16], particle size [17], pH [18], moisture content, solubility in water [14], solubility in 96% ethanol [14], molecular weight [19], deacetylation degree [20], loss of drying [43], ash content (SNI 01-2354.1-2006), heavy metal contamination [45], and microbial contamination [46].

### 2.6. Chitosan Depolymerization

Chitosan depolymerization was performed using a combination of weak acids, i.e., formic acid and ultrasonication. The chitosan was dissolved in 1% formic acid to obtain three concentrations, namely 0.5, 1, and 1.5%. Sonication was then performed on the chitosan solutions with temperature variations of 20, 40, and 60 °C and time variations of 10, 20, and 30 min (Table 1) using a sonicator (Kudos, Shanghai-China). Several studies have reported the effects of these conditions on the depolymerization of chitosan. The previous studies showed that chitosan was depolymerized faster in dilute acid solution and at lower temperatures [29]. It was reported that degradation of chitosan in very low concentration of weak acid [25]. The depolymerization temperature was varied at 40, 50, and 60 °C to study the influence of temperature on chitosan depolymerization, and the result showed that the molecular weight value was significantly reduced [27]. The degradation of chitosan was reported with the variations of sonication time of 30 to 120 min [25]. The depolymerization reaction was ceased by soaking the chitosan solution after the sonication was performed into ice. The samples were then collected and neutralized with 2 M NaOH until the pH reached 8–9. The solid formed was then filtered and washed with distilled water. Other contaminants were removed by rinsing the solids using 95% ethanol. The dried solids were then ground and characterized, including yield (%), degree of deacetylation, molecular weight using the viscometric method, structure analysis using FTIR and ^1^H-NMR, morphology, thermal analysis, and crystallinity index.

### 2.7. Optimization Process

Optimization of the depolymerization process was performed using the Box-Behnken Design using three independent variables, each of which used three levels (Table 2). There were 17 run orders with five center points, as shown in Table 3. The effects of each independent variable on the responses, i.e., molecular weight, degree of deacetylation, and yield, were measured using a second-order polynomial:

(1) $$y=\sum_{i=l}^{k}\beta oXi+\sum_{i=1}^{k}\beta iiXi^{2}+\sum_{i=1}^{k-1}\;\sum_{j=2}^{k}\beta ijXiXj+\;€\;$$

where *y* = predicted response, *βi* is a linear coefficient, *βii* is a quadratic coefficient, and *βij* is the interaction coefficient, *Xi*, *Xj*, and *Xk* are independent variables affecting the depolymerization processes, *ε* is random error. The success of the developed method was determined by coefficient *R^2^*. The significance of the regression coefficient model was evaluated using analysis of variance (ANOVA). After that, the 3-dimensional curve and response plot obtained were used to interpret the effect of the interaction between the independent variables on the response variables using design-expert version 13 [47].

### 2.8. Characterization of Low Molecular Weight Chitosan

#### 2.8.1. Fourier Transform Infra-Red (FTIR) Spectroscopy

The low molecular weight chitosan obtained was read using a FTIR spectrophotometer (Shimadzu^®^ IR-Prestige 21, Tokyo, Japan). The chitosan was made into pellets with KBr, then scanned at a frequency between 4000 cm^−1^ and 500 cm^−1^. The spectrum obtained was compared with those of chitin, initial chitosan, and standard chitosan [15,18].

#### 2.8.2. H-NMR Spectroscopy

^1^H-NMR spectral measurements were performed using a ^1^H-NMR spectrophotometer (JEOL, Peabody, MA, USA). Ten milligrams of chitosan were suspended in 7.5 mL of D2O containing two drops of trifluoro acetic acid (TFA). The mixture was vortexed for 3 m. The spectra between 0 and 15 ppm were recorded [48]. The acquisition time was 1.745 s and the delay time was 5 s. The field strength was 11.747 T, and the frequency was 500 MHz.

#### 2.8.3. Scanning Electron Microscope

The morphology of the obtained low molecular weight chitosan was observed under Scanning Electron Microscope (SEM) (Hitachi Series SU3500, Tokyo, Japan). A particular amount of chitosan was attached to the specimen holder with gold palladium plating, inserted into the specimen chamber, and run with a stereoscan microscope [49].

#### 2.8.4. Differential Scanning Calorimetry

This was analyzed using differential scanning calorimetry (DSC) ((NEXTA DSC, Tokyo, Japan), of which the temperature had been set and the cell constant used indium. A total of 1–3 g of chitosan was placed in an aluminum container and analyzed at 50–500 °C with a heating rate of 10 °C/min. The samples were cleaned continuously with nitrogen at 50 mL/min [26].

#### 2.8.5. X-ray Diffractometry

The crystallinity index was measured with XRD (X’Pert PRO PANatycal, Worcestershire, UK) using Cu-Kα radiation, where the detection was performed at 2θ = 5–50° at a speed of 2°/min and set at 40 kV and 30 mA. The chitosan powder was placed on the holder, and the crystallinity index was analyzed with the percentage of total area and crystal peak ratio [50].

## 3. Results and Discussion

### 3.1. Characterization of Shrimp Shell Powder

The result of the shrimp shell characterization is shown in Table 4.

### 3.2. Chitosan Characterization Based on Pharmaceutical Requirement

**Figure 2:**
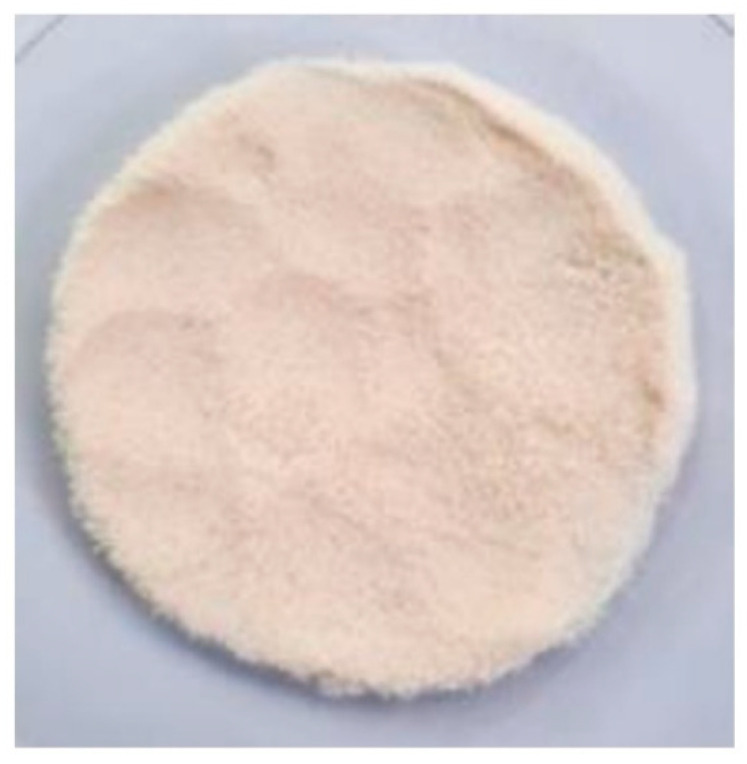
The isolated chitosan from vaname shrimp shell.

The isolated chitosan was presented in Figure 2. Chitosan characteristics based on pharmaceutical requirements are shown in Table 5.

The results of the characterization of chitosan isolated from vanname shrimps showed that the chitosan produced met the pharmaceutical grade requirements, as seen in Table 5. However, there was a difference with the pharmaceutical grade standard in terms of the solubility in water. The solubility of chitosan isolated from vaname shrimps was very slightly soluble, while the pharmaceutical grade standard has set that the solubility should be sparingly soluble. Such differences can be due to several factors. The factors that affect the solubility of chitosan include the degree of deacetylation, molecular weight, and crystallinity [51]. The degree of deacetylation of the chitosan produced (95.95%) was also higher than the standard degree of deacetylation. The degree of deacetylation is significantly affected by the preparation processes during chitosan isolation. A high degree of deacetylation is caused by prolonged deacetylation processes by alkali treatment of chitin with intermittent washing by water.

The chitosan isolated from vanname shrimp produced a yield of 26%. Yield is significantly influenced by the source of chitosan. The yield of chitosan obtained from shrimp shells in a previous study was reported to be 15.40% [52]. Meanwhile, the chitosan obtained from the *P.monodon* shrimp shell reached 35% [53]. The yield of chitosan obtained from crab shells was 30–32% [54]. The chitosan isolated from fungi was approximately 17.6% [55]. In addition, the chitosan isolated from fungi generated a yield of 1.5–3.5% [56]. The chitosan isolated from various species of insects also varied, ranging between 3.1%–96.2%. The yield of chitosan is also highly dependent on the variables of the isolation method used.

Chitosan identification using ninhydrin reagent showed a purple color after the chitosan was sprayed with 1% ninhydrin reagent. Ninhydrin is an oxidizing agent that reacts with the amino groups found in chitosan in a pH range of 4–8.

### 3.3. Depolymerization Process

Depolymerization of chitosan isolated from vanname shrimp was performed using a combination of formic acids and ultrasonication. The depolymerization process was optimized using the Box–Behnken design (BBD) with three variables and three levels (Table 1). The responses were in the form of chitosan molecular weight, degree of deacetylation, and yield, as shown in Table 6.

The results of the RSM calculation on the regression model of each predetermined parameter were seen from the lack of fit test. This model was used to determine and predict each response from each depolymerization parameter based on its significance. The accepted significance was *p* < 0.05. The lack of fit test results showed that the yield used the quadratic regression model while the molecular weight and degree of deacetylation used the linear regression. The ANOVA test showed that the regression model on the yield response could be used to determine the optimal condition of chitosan depolymerization with the combination of formic acids and ultrasonication methods. In contrast, the regression model for the other two responses could not be used. The response (yield) and the independent variables were correlated to a second order equation:


Yield (%) = 92.8 + 11.82A − 2.12B + 1.16 C + 8.68 AB − 2.63AC +1.60BC − 10.63A^2^ + 1.75B^2^ − 1.38 C^2^. (2)

**Figure 3:**
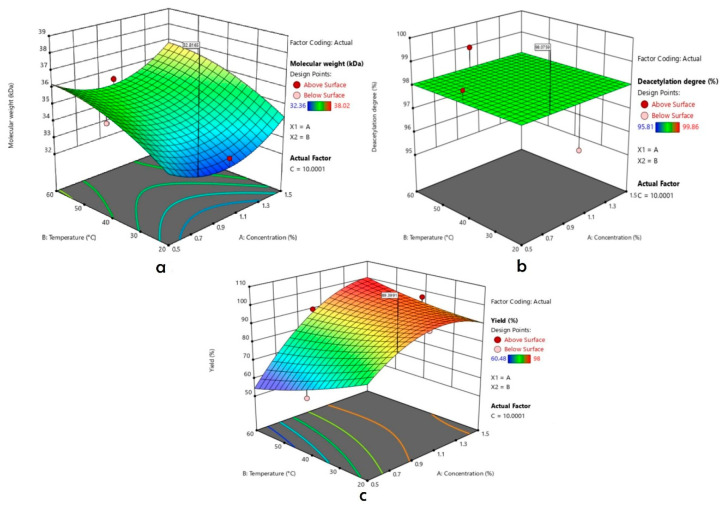
Three-dimensional diagram of (**a**) molecular weight, (**b**) deacetylation degree, and (**c**) yield prediction of the developed model.

The interaction between all factors was evaluated, and the results are shown in Figure 3.

The solution offered by the Box–Behnken design for the depolymerization process was to use three variables, namely concentration, temperature, and time, i.e., 0.733%, 20 °C, and 10 min. The predicted responses in terms of molecular weight, degree of deacetylation, and yield were 32.814 kDa, 98.076%, and 89.398% respectively.

The verification of the optimum conditions recommended by the Box–Behnken design shows that the molecular weight, deacetylation degree, and yield values are consistent with the predicted values. The molecular weight, deacetylation degree, and yield values of low molecular weight chitosan at optimum conditions were 32.359 kDa, 98.86%, and 89.33%.

The optimal conditions were then used to make low molecular weight chitosan further characterized, including structure, particle shape and surface observation, thermal analysis and measurement of crystallinity index.

### 3.4. Low Molecular Wight Chitosan Characterization

#### 3.4.1. Fourier Transform Infra-Red (FTIR) Spectroscopy

**Figure 4:**
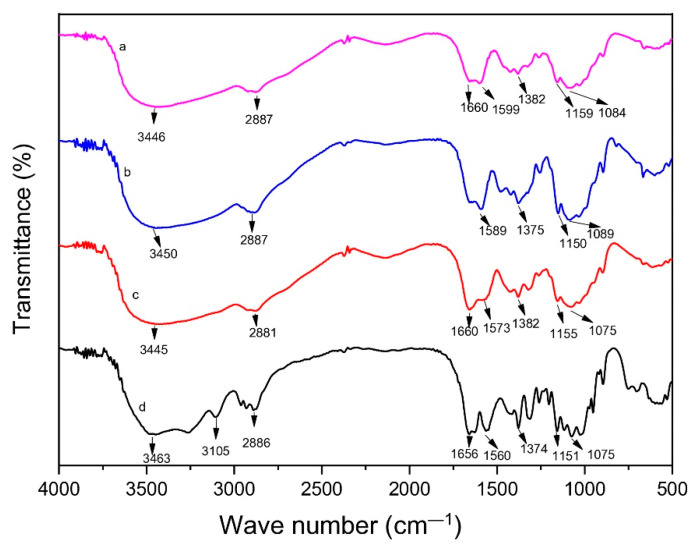
FTIR spectrum of low molecular weight chitosan (**a**), native chitosan (**b**), commercial chitosan (**c**), and (**d**) chitin.

The FTIR spectrum of low molecular weight chitosan, native chitosan, commercial chitosan, and chitin is shown in Figure 4.

The spectrum of low molecular weight chitosan was quite similar to native chitosan, indicating that the chitosan structure remains stable after the depolymerization process. A band around 3500 cm^−1^ in the FTIR spectra of low molecular weight chitosan and native chitosan indicates the presence of OH group. The band around 2887 cm^−1^ is attributed to C-H stretching. The band around 1660 cm^−1^ in the FTIR spectra of low molecular weight chitosan indicates the presence of the C=O group. The intensity of this peak was decreased compared to the native chitosan due to the decrease of acetyl group in low molecular weight chitosan compared to native chitosan. The presence of a band in 1559 cm^−1^ corresponds to N-H bending group. The bands around 1382 cm^−1^, 1159 cm^−1^ and 1084 cm^−1^ were attributed to C-O, C-N, and C-C groups, respectively. The results obtained were also compared with the commercial chitosan and chitin. Compared to FTIR spectra of chitin, there was a decrease of intensity peak around 1660 cm^−1^ due to the decrease of acetyl content because of the deacetylation process performed in chitin.

#### 3.4.2. H-NMR Spectroscopy

**Figure 5:**
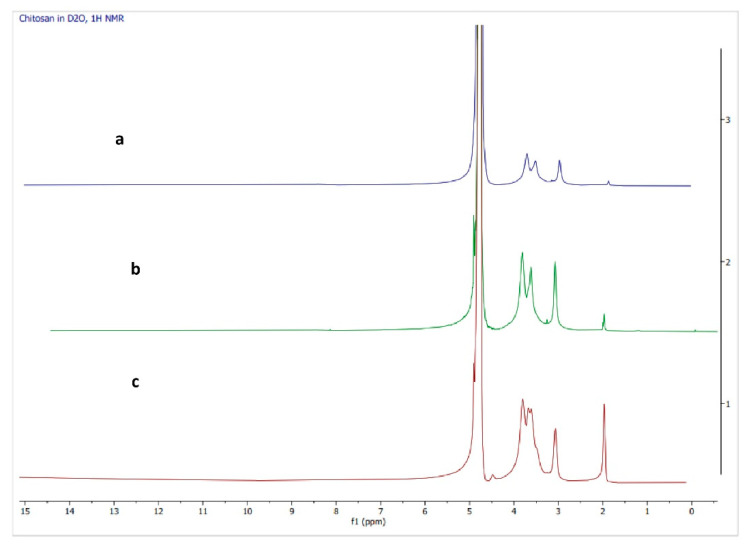
NMR spectrum of: (a) LMW chitosan, (b) native chitosan; (c) commercial chitosan.

NMR spectra of low molecular weight chitosan, native chitosan, and commercial chitosan are shown in Figure 5.

Chitosan, *N*-glucosamine, is a result of deacetylation reaction of chitin. The basic structure of chitosan is similar to that of chitin, except that the acetyl groups in chitin have been partially removed to give an amine (NH_2_) group, thus constructing a polymer that comprises both *N*-glucosamine and *N*-acetylglucosamine as the monomers that make up the building block. The result of a conversion from chitin to chitosan through deacetylation reaction can be observed in an NMR spectrum. As in the case of chitin, the NMR spectrum of chitosan should show similar signals pattern. The only striking difference between chitin and chitosan in NMR spectrum lies in the presence of methyl proton signals. In chitin, the methyl signals should appear prominently at around 2.0 ppm. However, these signals should decrease in chitosan as some portions of the acetyl groups have been removed. The NMR spectrum of the native chitosan (Figure 5b) shows a signal pattern that corresponds to how a chitosan NMR spectrum should be performed. It can be observed in the spectrum that the methyl signals (around 2.0 ppm) are significantly lower than the other signals. The spectrum is comparable to commercial chitosan (Figure 5c), which exhibits similar signal patterns, suggesting a success in the deacetylation process that resulted in the native chitosan. Furthermore, the results of a depolymerization reaction carried out on the native chitosan to produce a low molecular weight chitosan (Figure 5a) indicated the presence of a product with a similar signal pattern in ^1^H NMR. However, the chain shortening needs to be observed using MS data and cannot be confirmed with NMR spectroscopy alone.

#### 3.4.3. Scanning Electron Microscope

**Figure 6:**
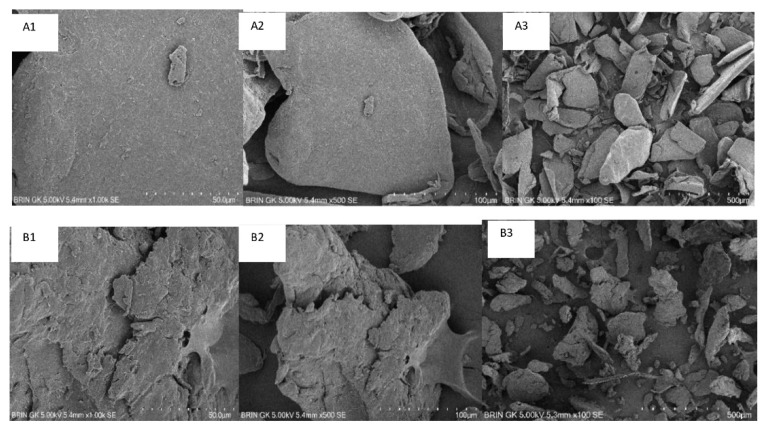
SEM of native chitosan magnification at 1000× (**A1**), 500× (**A2**), 100× (**A3**) and SEM of low molecular weight chitosan magnification at 1000× (**B1**), 500× (**B2**), 100× (**B3**).

The morphology of the obtained low molecular weight chitosan and native chitosan is shown in Figure 6.

Observation using SEM showed that the low molecular weight chitosan had a more disorganized shape compared to native chitosan. The observation of the native chitosan showed an irregular shape, fibrous network, and smooth surface.

#### 3.4.4. Thermal Analysis Using Differential Scanning Calorimetry

**Figure 7:**
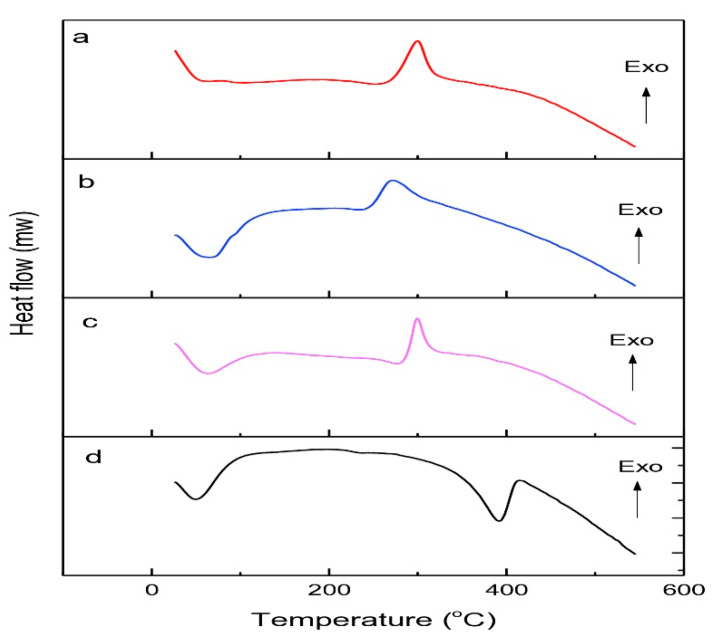
DSC thermogram of (**a**) low molecular weight chitosan, (**b**) native chitosan, (**c**) comercial chitosan, and (**d**) chitin.

Thermal analysis using DSC is shown in Figure 7.

Thermal analysis using DSC on low molecular weight chitosan, native chitosan, and commercial chitosan showed a broad endothermic peak around 60–65° related to water dehydration. The exothermic peak of chitosan correspondent to amine group (GLcN) decomposition. Meanwhile, the DSC curves of chitin showed two endothermic peaks, whereas the peak around 50 °C correspondents to water loss, and the peak around 392 °C is related to chitin degradation. The endothermic enthalpies energy of low molecular weight of chitosan was decrease compared to native chitosan, indicating that these chitosan molecules differ in their strength of chitosan-water interaction and water-chitosan bonding capacity. Furthermore, the increase of exothermic enthalpies of low molecular weight chitosan compared to native chitosan indicates that these molecules differ in their strength of intramolecular bonding capacity due to the chain shortening.

#### 3.4.5. X-ray Diffractometry

**Figure 8:**
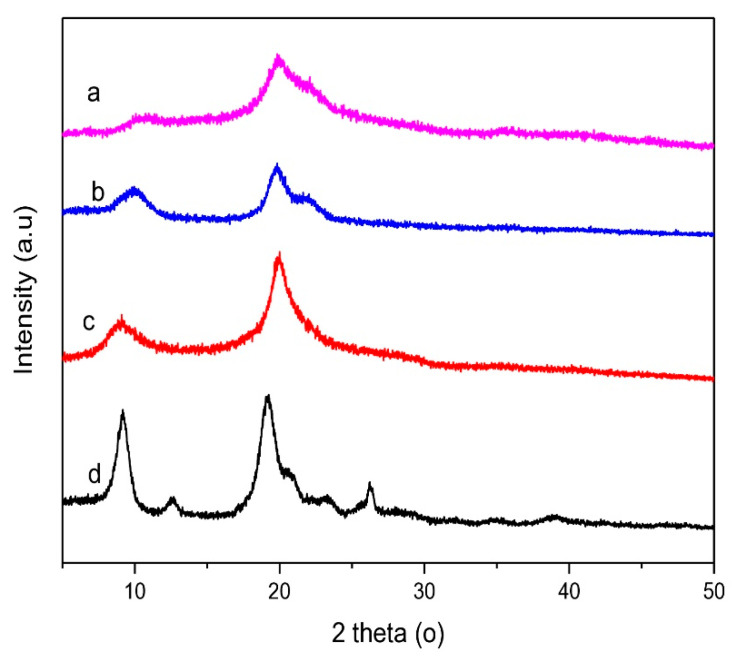
Diffractogram of (**a**) low molecular weight chitosan, (**b**) native chitosan, (**c**) commercial chitosan and (**d**) chitin.

The crystallinity index and diffractogram of low molecular weight chitosan, native chitosan, commercial chitosan, and chitin are shown in Table 7 and Figure 8, respectively.

The diffractogram of low molecular weight chitosan showed how a chitosan diffractogram should be performed. There were diffraction peaks at 2θ = 10.62° and 2θ = 19.86°. The diffractogram was comparable to that of native chitosan and commercial chitosan, which exhibits a similar pattern. The intensity of low molecular weight chitosan diffraction peaks decreases correspondents to partial decrystallization of the chitosan. Crystallinity index decreased in the following order: low molecular weight chitosan > native chitosan > chitin due to increasing amorphous domains of chitosan. The increasing of amorphous domains of chitosan is correspondent to the sorption ability. The sorption mechanism is absorption of water molecules into the hydrophilic domain of amorphous chitosan [57].

## 4. Conclusions

The production of low molecular weight chitosan involving depolymerization in the presence of weak acid and ultrasonication can be developed commercially to produce low molecular weight chitosan. The optimal depolymerization process was studied by Box–Behnken design (BBD). The optimal condition and the best responses were obtained. The optimum concentration was 0.733%, the optimum temperature was 20 °C, and the optimum sonication time was 10 min. IR and NMR spectrum confirmed that there was no modified chemical structure of low molecular weight chitosan compared to native chitosan. There was no thermal behavior transformation in low molecular weight compared to native chitosan. The reduction of molecular weight of chitosan led to an increase in the amorphous domain of chitosan. However, data regarding the toxicity of low molecular weight of chitosan obtained by this method need to be evaluated because the chitosan will be applied in the future in pharmaceutical applications.

## Figures and Tables

**Table 1:** The materials of research.

No	Materials	Source/Manufacturer
1	Vaname shrimp shell	PT Yanagi (Kendari, Indonesia)
2	Commercial chitosan	Sigma-Aldrich (Saint.Louis, MO, USA)
3	Formic acid	CV Multi Usaha Mandiri (Sidoarjo, Indonesia)
4	Acetic acid	CV Sumber Rejeki (Makassar-Indonesia)
5	Sodium hydroxide	CV Sumber Rejeki (Makassar-Indonesia)
6	Hydrochloric acid	CV Sumber Rejeki (Makassar-Indonesia)
7	ethanol 96%	CV Sumber Rejeki (Makassar-Indonesia)
8	deionized water	CV Sumber Rejeki (Makassar-Indonesia)
9	Potato Dextrose Agar	Merck KGaA (Darmstads, Germany)
10	*Staphilococcus aureus* ATCC25923	Laboratory of Biomedic, Faculty of Medicine, Halu Oleo University, Kendari, Indonesia
11	*Escherichia coli* ATCC 35218	Laboratory of Biomedic, Faculty of Medicine, Halu Oleo University, Kendari, Indonesia
12	*Salmonella sp*. ATCC 14028	Laboratory of Biomedic, Faculty of Medicine, Halu Oleo University, Kendari, Indonesia

**Table 2:** Level of experimental variables.

Level	Chitosan Concentration (%)	Temperature (°C)	Time (Minute)
Low (−1)	0.5	20	10
Middle (0)	1	40	20
High (+1)	1.5	60	30

**Table 3:** Box Bhenken design for three factors and three levels.

No	Run Order	Chitosan Concentration (%)	Temperature (°C)	Time (Minute)
1	11	0	−1	+1
2	7	−1	0	+1
3	8	+1	0	+1
4	16	0	0	0
5	14	0	0	0
6	6	+1	0	−1
7	2	+1	−1	0
8	15	0	0	0
9	17	0	0	0
10	9	0	−1	−1
11	12	0	+1	+1
12	13	0	0	0
13	3	−1	+1	0
14	4	+1	+1	0
15	10	0	+1	−1
16	1	−1	−1	0
17	5	−1	0	−1

**Table 4:** Characteristics of shrimp shell powder.

Specification	Shrimp Shell Powder
Organoleptic	Coarse powder, slightly yellowish, strong shrimp shell odour, less tasty
Solubility in water	5%
Solubility in 96% ethanol	3%
Ash content	14.14%
Water content	54.57%
Fat content	1.81%
Protein content	14.92%

**Table 5:** Chitosan characteristics are based on the pharmaceutical requirement.

Specification	Isolated Chitosan	Pharmaceutical Specification of Chitosan	Reference
Organoleptic properties	Odourless, creamy white powder	Odourless, white or creamy white, or flakes	Rowe, 2009
Particle size	4.3702 µm (PI = 0.679)	<30 µm	Rowe, 2009
pH	4.68	4.0–6.0	Rowe, 2009
Moisture content	5.09%	≤5%	USP-36, NF-31
Solubility:			PhEur-5
Water	Very slightly soluble	Sparingly Soluble	
Ethanol	Practically Insoluble	Practically Insoluble	
Molecular weight	57,543.99 Dalton	≤1,000,000	USP-36, NF-31
Degree of deacetylation	95.95%	70–95%	USP-36, NF-31
Loss on drying	0.37%	≤10%	Rowe, 2009
Ash content	0.87%	<1%	Rowe, 2009
Heavy metal Lead (Pb) Cadmium (Cd) Mercury (Hg)	0.0002 mg/L 0.0004 mg/L <0.0001 mg/L	≤40 ppm	Rowe, 2009
Microbial contamination			SNI, 2013
*Escherecia coli*	<3 MPM/g	<3 MPM/g	
*Salmonella*	-		
Total plate count (Bacteria)	270 colony/g	Maximum 1 × 10^3^ colony/g	
Total plate count (fungi)	350 colony/g		
Yield	26%		
Nynhidrine test	Purple		

**Table 6:** Box Bhenken Design and Result.

No	Factor			Response		
	Concentration (%)	Temperature (°C)	Time (Minute)	Molecular Weight (kDa)	Deacetylation Degree (%)	Yield (%)
1	1	20	30	35.48	96.69	92.90
2	0.5	40	30	35.48	99.44	69.68
3	1.5	40	30	38.02	99.01	95.4
4	1	40	20	36.31	97.85	84.5
5	1	40	20	33.88	99.13	98
6	1.5	40	10	36.67	96.77	96.70
7	1.5	20	20	36.3	99.61	86.20
8	1	40	20	34.67	95.81	88.10
9	1	40	20	34.67	96.82	94.30
10	1	20	10	33.11	96.16	95.40
11	1	60	30	32.36	96.70	93.70
12	1	40	20	33.88	99.27	98
13	0.5	60	20	34.67	99.86	63.84
14	1.5	60	20	36.31	97.86	97.50
15	1	60	10	35.48	98.96	89.8
16	0.5	20	20	34.67	98.76	87.28
17	0.5	40	10	35.11	98.59	60.48

**Table 7:** Crystallinity Index Value.

Samples	The Cristalinity Index (%)
Low molecular weight chitosan	36.64
Native chitosan	42.01
Commercial chitosan	42.90
Chitin	61.29

## Data Availability

The data presented in this study are available on request from the corresponding author.
